# Detecting the Diversity of *Mycoplasma* and *Ureaplasma* Endosymbionts Hosted by *Trichomonas vaginalis* Isolates

**DOI:** 10.3389/fmicb.2017.01188

**Published:** 2017-06-28

**Authors:** Anastasios Ioannidis, Panagiota Papaioannou, Emmanouil Magiorkinis, Maria Magana, Vasiliki Ioannidou, Konstantina Tzanetou, Angeliki R. Burriel, Maria Tsironi, Stylianos Chatzipanagiotou

**Affiliations:** ^1^Department of Nursing, Faculty of Human Movement and Quality of Life Sciences, University of PeloponneseSparta, Greece; ^2^Department of Biopathology and Clinical Microbiology, Athens Medical School, Aeginition HospitalAthens, Greece; ^3^Department of Hygiene, Epidemiology and Medical Statistics, Medical School, University of AthensAthens, Greece; ^4^Department of Clinical Microbiology, “Alexandra” General HospitalAthens, Greece

**Keywords:** *Trichomonas vaginalis*, *Mycoplasma*, *Ureaplasma*, *Candidatus Mycoplasma girerdii*, phylogeny, endosymbiosis, trichomoniasis

## Abstract

**Objectives:** The symbiosis of *Trichomonas vaginalis* and *Mycoplasma hominis* is the first described association between two obligate human parasites. *Trichomonas* is the niche and the vector for the transmission of *M. hominis* infection. This clinically significant symbiosis may affect *T. vaginalis* virulence and susceptibility to treatment. The aims of this study were to investigate the intracellularly present *Mycoplasma* and *Ureaplasma* species in *T. vaginalis* strains isolated from the vaginal discharge of infected women as well as to trace the diversity pattern among the species detected in the isolated strains.

**Methods:** Hundred pure *T. vaginalis* cultures were isolated from ~7,500 patient specimens presented with clinical purulent vaginitis. PCR and sequencing for *Mycoplasma/Ureaplasma* spp. were performed in DNA extracted from the pure cultures. In addition, vaginal discharge samples were cultured for the presence of *M. hominis* and *U. urealyticum*. Phylogenetic analysis assisted the identification of interspecies relationships between the *Mycoplasma* and *Ureaplasma* isolates.

**Results:** Fifty four percentage of *T. vaginalis* isolates were harboring *Mycoplasma* spp. Phylogenetic analysis revealed three distinct clusters, two with already characterized *M. hominis* and *Ureaplasma* spp. (37% of total *Mycoplasma* spp.), whereas one group formed a distinct cluster matched with the newly identified species *Candidatus Mycoplasma girerdii* (59.3%) and one or more unknown *Mycoplasma* spp. (3.7%).

**Conclusions:** *T. vaginalis* strains associated with vaginal infection might host intracellular mycoplasmas or ureaplasmas. Intracellular Mollicutes that remain undetected in the extracellular environment when conventional diagnostic methods are implemented may comprise either novel species, such as *Candidatus M. giredii*, or unknown species with yet unexplored clinical significance.

## Introduction

The flagellated protozoan parasite *Trichomonas vaginalis* is one of the most common causes of non-viral sexually transmitted infections in humans, annually affecting at least 142 million people worldwide (WHO, 2016). Most *Trichomonas* spp., other than *T. vaginalis*, are harmless commensals, colonizing the gastrointestinal tract of a broad variety of hosts (Soper, 2004). The majority of infected individuals remains asymptomatic (up to 85%) and the infection might persist for months or years (Sutton et al., 2007; Kissinger, 2015). Clinical manifestations mainly occur in women and vary from mild to severe vaginitis with profuse inflammatory discharge. The course of the infection depends on the individualized host defense mechanisms and the virulence factors of the causative strain (Arroyo et al., 1992; Mendoza-Lopez et al., 2000). Strains of *T. vaginalis* also present variable susceptibility profiles toward metronidazole and tinidazole, the major antitrichomonal drugs (Sena et al., 2014). Therefore, imponderable etiologic factors along with the reported paucity of therapeutic options impede the treatment of vaginitis.

The clinical importance of the *T. vaginalis* infections has been reportedly associated with dysbiosis of the urogenital tract microbiome and with the co-existence of the protist with opportunistic microorganisms including the intracellularly hosted *Mycoplasma* spp. or the *T. vaginalis* viruses (TVVs; Bar et al., 2015; Margarita et al., 2016). The *Trichomonas* viruses constitute the first double-stranded RNA (dsRNA) viruses associated with the parasite, and the notion that the trichomonads infected by the TVVs may present prolonged survival within the human host due to immunogenic proteins expression modulation is not new (Goodman et al., 2011).

The partnership between the protozoan and the bacteria or the viruses exerts a modulatory effect on innate immunity and the inflammatory process (Rappelli et al., 2001; Hirt and Sherrard, 2015). *T. vaginalis* and *M. hominis* are known to have the same arginine dihydrolase (ADH) pathway, which serves as an alternative source of ATP through arginine scavenging. Subsequent arginine depletion blocks nitric oxide (NO) production by host macrophages, alters host defense mechanism and ameliorates the parasite survival (Morada et al., 2010). Furthermore, the extensively studied symbiotic pattern of *M. hominis* hosted by *T. vaginalis* is proved to play a key role in inflammation during trichomoniasis, thus enhancing the severity of the disease (Dessi et al., 2005, 2006; Vancini and Benchimol, 2008; Vancini et al., 2008; Morada et al., 2010; Fiori et al., 2013). *T. vaginalis* hosting *M. hominis* have also been found to bear higher phagocytic activity than *Mycoplasma*–free isolates (Cirillo et al., 1997).

The concept of reductive evolution of the mycoplasmal genome from Gram-positive bacteria, based on comparative genomics, justifies both structural and functional alterations (Razin et al., 1998). *Mycoplasma* species, belonging in the class of Mollicutes, have developed a small genome which lacks biosynthetic pathways involved in macromolecule building blocks synthesis, therefore explaining the absence of cell wall (Liu et al., 2012). The *Mycoplasma* genome also carries a minimal set of energy metabolic genes with a restricted adenosine triphosphate (ATP) supply which is essential for their parasitic mode of life (Razin, 1997). Despite the limited genetic redundancy, *Mycoplasma* spp. present a rather interesting mechanism of self-protection based on their ability to modulate the inflammatory response of the host immune system (Razin et al., 1998).

Among *Mycoplasma* spp., *M. hominis* is the most frequently isolated microorganism from the genital tract of both males and females, acting either as part of normal flora or as a putative pathogen responsible for a variety of urogenital infections (Ladefoged, 2000). The Mollicutes represent the most common pathogens associated with *T. vaginalis* infection with *Mycoplasma hominis* and *Candidatus Mycoplasma girerdii* as the predominant strains isolated in trichomoniasis specimens (Koch et al., 1997; Fettweis et al., 2014). The symbiotic phenomenon of *M. hominis* hosted by the pathogenic protist has received special interest though little is known about the intracellular colonization of *T. vaginalis* by other *Mycoplasma* spp. (Martin et al., 2013). The study of the symbiotic relationship between *T. vaginalis* and *M. hominis* provides information about (i) the association between two obligate human pathogens and (ii) an additional role of *T. vaginalis* as a novel niche for *M. hominis*. Infected *T. vaginalis* isolates act as *M. hominis* carriers mediating transmission in other protozoa and human-derived epithelial cells (Rappelli et al., 2001). Mycoplasmas hosted by *T. vaginalis* have the privilege to evade host immune response, enhance *T. vaginalis* virulence and affect susceptibility to antibiotic treatment, thus complicating the infection eradication process (Dessi et al., 2005). In particular, a higher rate of phagocytic activity and amoeboid transformation, indicative of increased virulence, is observed in *T. vaginalis* isolates harboring *M. hominis* as compared with the mycoplasma-free isolates (Vancini et al., 2008). The endosymbiotic relationship may also alter the host inflammatory respond during trichomoniasis subsequently affecting disease severity (Fiori et al., 2013).

The broad term “mycoplasmas” collectively refers to both the *Mycoplasma spp*. and the *Ureaplasma spp*. The latter differentiate to *Ureaplasma urealyticum* and *Ureaplasma parvum* which mainly colonize the urogenital tract mucosal surfaces of sexually active women (McIver et al., 2009). *U. urealyticum* used to be categorized into biovars 1 and 2; however, according to phylogenetic analysis this taxonomic overlap has changed with the identification of the *U. parvum* formerly known as *U. urealyticum* biovar 1 (Kong et al., 1999). Both species have been controversial for their participation in human diseases and are generally considered as “low-grade pathogens” (Cox et al., 2016). The arrival of *U. parvum* at the forefront of research studies has raised questions regarding the virulence and pathogenicity of the microorganism formerly known as a non-pathogenic commensal of the male urethra. In fact, recent studies have proved the participation of the bacterium in pregnancy complications, intrauterine infections, vaginosis, and pelvic disease (McIver et al., 2009; Larsen and Hwang, 2010; Cox et al., 2016). Of great clinical significance is the association of preterm neonates ureaplasmal infection with adverse health outcomes predominantly including bronchopulmonary dysplasia, central nervous system deformities, and necrotizing enterocolitis (Pandelidis et al., 2013; Viscardi, 2014; Glaser and Speer, 2015). To date, Mollicutes including both *Mycoplasma* and *Ureaplasma* spp. are known to actively participate in sexually-transmitted infections though the exact role of the latter in trichomonas-associated infections is not widely investigated.

In this study the intracellular presence of *Mycoplasma* and *Ureaplasma* species was investigated employing *T. vaginalis* strains isolated from infected women with vaginitis. The detected bacteria were identified and classified, aiming to disclose whether species other than *M. hominis* can endosymbiotically colonize *T. vaginalis*. Ultimately, a major aspect of this study was to surveil both the diversity and the genetic distance among the species of the isolated strains.

## Methods

### Patients

A total number of 7,500 clinical samples collected from adult women aged 18–64 years was incorporated in this study within a 3-year period (2010–2012). A written informed consent is not required since the clinical isolates do not provide identifying information regarding the participants, as it is in the present study. All patients were presented with purulent vaginitis at the outpatient clinic of the Department of Gynecology of “Alexandra” General Hospital in Athens. From the total samples number, only 100 were confirmed as *T. vaginalis* positive containing the parasite and were further cultured to investigate the presence of *Mycoplasma/Ureaplasma* spp.

### Sample collection and microbiological processing

Four vaginal swab specimens were collected from each patient for: (i) microscopic examination, (ii) conventional bacteriological culture, (iii) *T. vaginalis* culture, and (iv) *Mycoplasma/Ureaplasma* detection. *T. vaginalis* strains were cultured by daily passages in commercially available Trichosel broth, a modified version of the Simplified Trypticase Serum (STS) Medium (Becton, Dickinson and Company, New Jersey, USA; Kupferberg et al., 1948). Specimens detected to contain *T. vaginalis* through microscopy, were inoculated into the broth and incubated at 37°C in an aerobic atmosphere. Growth was observed after 2 and 5 days of incubation by wet mount microscopy, and positive samples were subcultured in fresh Trichosel medium in order to enrich the parasite number. The medium contained Chloramphenicol (0.1 g/L) for bacterial growth inhibition including *Mycoplasma*/*Ureaplasma*. The initial cultures were followed by four subcultures before harvesting the parasites for further processing. Both primary cultures and subcultures were tested for the presence of extracellular mycoplasmas; an aliquot of each *T. vaginalis* culture was centrifuged at 500 g for 10 min, and the supernatants were filtered through a 0.45 μm-pore-size filter membrane that only allows small microbial cells such as mycoplasmas to pass through (Dutscher Scientific; Essex, UK). The filtrate was used for DNA extraction and subsequent polymerase chain reaction (PCR) for the detection of *Mycoplasma*/*Ureaplasma* spp. Sediments rich in *T. vaginalis* were stored at −80°C for further handling.

*Mycoplasma/Ureaplasma* spp. detection in vaginal swabs was performed using conventional bacteriological procedures. Swabs were inoculated in a commercial broth for the detection of *M. hominis* and *U. urealyticum* (Mycoplasma U-A broth, CE marked, Bioprepare Microbiology, Attica, Greece). Differential identification was based on a semi-quantitative test fermentation test. More specifically, the kit contains two tubes, one for the *M. hominis* discrimination based on the arginine fermentation, and one for the *U. urealyticum* discrimination based on the urea fermentation. The identification of the presence of the *Mycoplasma* or *Ureaplasma* spp. is based on the arginine and urea fermentation process leading to an alkaline pH shift and a respective color change from orange to red in the corresponding tube. Broth with a positive reaction was further plated on a commercially available selective medium (Mycotest agar, CE marked, Bioprepare Microbiology, Attica, Greece) that provides all the nutrients needed for the growth of *Mycoplasma* species along with the appropriate pH value. It also contains antibiotics and antifungal agents that inhibit the growth of most bacteria and fungi. Identification was performed by microscopic characterization of the typical colonies.

### DNA extraction

The DNA extraction from the *T. vaginalis* rich Trichosel broth culture sediments and the filtered supernatants was performed with the commercially available extraction reagent “Instagene Matrix” (Bio-Rad Laboratories, California, U.S.A.), according to the manufacturer's instructions.

### PCR amplification for *T. vaginalis* and *Mycoplasma/Ureaplasma* spp.

The commercial ready mix “Jumpstart Red Taq” (SIGMA Laboratories, St. Louis, U.S.A.) was used for the PCR amplification. The final mix contained 25 μL Jumpstart Red Taq, 1 μM of each primer, 20 μL distilled water and ~300 ng template. Primers for *T. vaginalis* (TVK-3 and TVK-7) and for *Mycoplasma/Ureaplasma* (GPO-1 and MGSO) were provided by Invitrogen (Primer design Ltd, Southampton, United Kingdom) according to previously reported design (van Kupperveld et al., 1992; Pillay et al., 2007). The primer final concentration was 10 pmol/μL for each PCR reaction targeting *T. vaginalis* detection. An aliquot of initial PCR sample was analyzed by conventional 2% agarose gel electrophoresis. DNA was stained with ethidium bromide, visualized and photographed with a computerized transilluminator system (Chemidoc, Bio-Rad Laboratories, California, U.S.A.). Primers and reaction profiles are shown in Tables 1, 2. In positive samples, a distinct 261 bp band was detected for *T. vaginalis* and a 717 bp band for *Mycoplasma*/*Ureaplasma*.

**Table 1:** Primers and reaction profile for *Trichomonas vaginalis* (Pillay et al., 2007).

**Primer**	**Target gene**	**Sequence (5′ → 3′)**	**Product (bp)**
TVK-3	*T. vaginalis*-specific repeat DNA fragment	ATTGTCGAACATTGGTCTTACCCTC	261
TVK-7		TCTGTGCCGTCTTCAAGTATGC	

*Reaction profile: 1 cycle → 94°C for 1 min; 35 cycles → 94°C for 1 min, 60°C for 30 s, and 72°C for 1 min; 1 cycle → 72°C for 5 min; 1 cycle → 0°C for ∞*.

**Table 2:** Primers and reaction profile for *Mycoplasma/Ureaplasma* spp. (van Kupperveld et al., 1992).

**Primer**	**Target gene**	**Sequence (5′ → 3′)**	**Product (bp)**
GPO-1	*Mycoplasma* genus-specific 16S rRNA	ACTCCTACGGGAGGCAGCAGTA	717
MGSO		TGCACCATCTGTCACTCTGTTAACCTC	

*Reaction profile: 1 cycle → 94°C for 1 min; 35 cycles → 94°C for 1 min, 55°C for 1 min and 72°C for 1 min; 1 cycle → 72°C for 5 min; 1 cycle → 0°C for ∞*.

### Quantitative PCR for *Mycoplasma/Ureaplasma* spp.

For the exclusion of the mycoplasmal DNA presence in the filtered supernatant, a protocol for DNA amplification was performed with a TaqMan probe for *M. hominis, M. genitalium, U. urealyticum* and *U. parvum* as described previously (Olsen et al., 2009; Ferandon et al., 2011; Frolund et al., 2014). Primers and reaction profiles are summarized in Table 3. All tests were performed in duplicates with ~300 ng DNA template. Different quantitative real-time PCR standard curves were generated by analyzing 10-fold serial dilutions of purified DNA from *M. hominis, M. genitalium, U. urealyticum*, and *U. parvum* as previously described (Campos et al., 2015).

**Table 3:** Primers and reaction profile for quantitative PCR of *M. hominis* and *M. genitalium, U. urealyticum* and *U. parvum* (Olsen et al., 2009; Ferandon et al., 2011; Frolund et al., 2014).

**Primer**	**Target gene**	**Sequence (5′ → 3′)**	**Species**
MHyidCfwd	*yidC*	TCACTAAACCGGGTATTTTCTAACAA	*M. hominis*
MHyidCrev		TTGGCATATATTGCGATAGTGCTT	
Probe MHyidC		FAM-CTACCAATAATTTTAATATCTGTCGGTATG-MGB	
MgPa-355F	*MgPa*	GAGAAATACCTTGATGGTCAGCAA	*M. genitalium*
MgPa-432R		GTTAATATCATATAAAGCTCTACCGTTGTTATC	
Probe MgPa-380	Urease gene	FAM-ACTTTGCAATCAGAAGGT-MGB	*U. urealyticum*
U195F		GCAAGAAGACGTTTAGCTAGAGGTTT	
U8R		CACGAGCAGATTGCATTAAGTCAG	
Probe U8		FAM-TAATTACTGACCACGTAGTGGA-MGB	
U195F	Urease gene	GCAAGAAGACGTTTAGCTAGAGGTTT	*U. parvum*
U3R		CGAGCAGATTGCATTAGGTCAG	
Probe U3		FAM-TTTAATTACTGATCATGTAATGGA-MGB	

*Reaction profile: 1 cycle → 50°C for 1 min 94°C for 10 min; 45 cycles → 95°C for 15 s, 60°C for 1 min and 72°C for 1 min*.

### Sequence analysis

Capillary Sanger/dideoxy labeling was performed for the sequencing of the PCR products by Macrogen Incorporation (908 World Merdian Center #60–24 Gasan-dong, Geumchum-gu Seoul, Republic of Korea).

### Phylogenetic analysis of *Mycoplasma/Ureaplasma* spp.

Species definition was accomplished through the phylogenetic analysis of the sequenced samples and the investigation of the phylogenetic relationship between different isolates within the same species. 16S rRNA sequences from each *Mycoplasma* and *Ureaplasma* spp. were downloaded (available at GenBank database until March 2015) reaching a total of 302 sequences representing 184 different *Mycoplasma* species, six different *Ureaplasma* species and several uncultured and unclassified *Mycoplasma* and *Ureaplasma* species. Genetic and phylogenetic analysis was performed with MEGA software (MEGA v6.0), whilst sequences were aligned using the “Muscle” algorithm and were manually edited (Edgar, 2004; Tamura et al., 2013). Genetic distances between the sequences were calculated using the Tamura-Nei model (Tamura and Nei, 1993; Tamura et al., 2011). Phylogenetic trees were constructed using the neighbor-joining method and their reliability was tested by bootstrapping analysis (1,000 replicates) (Saitou and Nei, 1987; Tamura et al., 2011). One cluster was considered significant if it was present in more than 75% of the permuted trees.

## Results

### Molecular detection

54% of *T. vaginalis* isolates were harboring *Mycoplasma* species. Vaginal discharge cultures revealed one sample infected with *M. hominis* and one with *U. urealyticum* (Table 4). All filtered supernatants from *T. vaginalis* broth cultures, including the two positive vaginal specimens, were negative in quantitative Real-time PCR tests for the presence of *M. hominis, M. genitalium, U. urealyticum*, and *U. parvum* DNA.

**Table 4:** *Mycoplasma/Ureaplasma* spp. intracellularly detected in *T. vaginalis* isolated from vaginal discharge of infected women by PCR, as well as culture results of the corresponding clinical material.

	***T. vaginalis*** **(*****n*** = **100)**		
	**Intracellular strains by PCR**	**Vaginal discharge culture**	
		**Positive**	**Negative**
*M. hominis*	14	1	13
*Mycoplasma* spp.	2	–	2
*Candidatus M. girerdii*	32	–	32
*U. urealyticum*	1	1	–
*U. parvum*	5	–	5
Negative for Mollicutes	46	–	46
Total	100	2	98

### Isolates identification

Sequence analysis of *T. vaginalis* PCR product confirmed the identification of the parasite in all isolates. Sequence analysis of the 54 mycoplasmal PCR products isolated from *T. vaginalis* revealed 14 *M. hominis* (25.9%), 1 *U. urealyticum* (1.9%), 5 *U. parvum* (9.3%), 32 *Candidatus Mycoplasma girerdii* (59.3%), and 2 unknown *Mycoplasma* spp. (3.7%) strains (Table 4).

### Species classification

**Figure 1:**
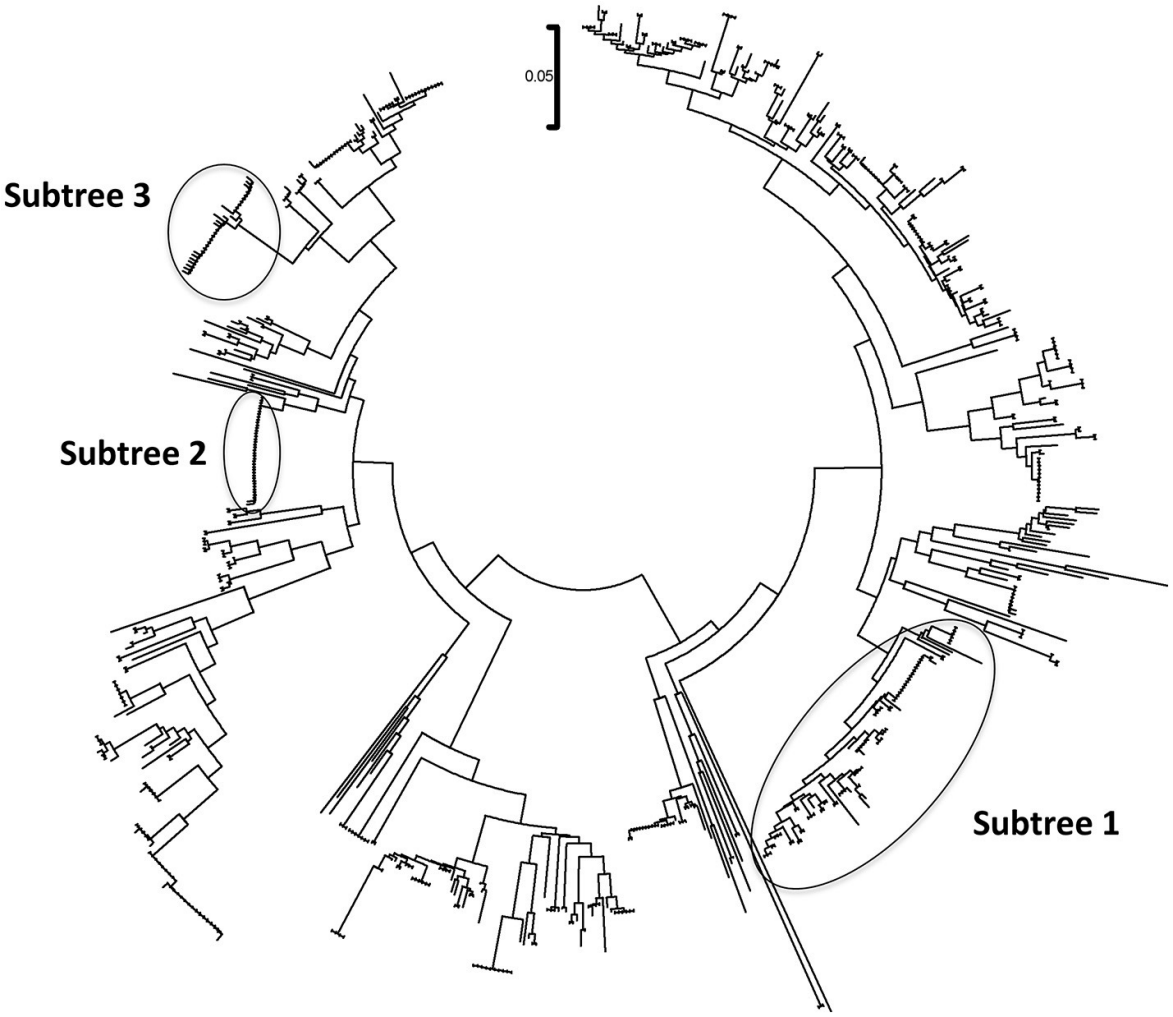
Circular phylogenetic tree including all detected and reference sequences. The evolutionary history was inferred using the Neighbor-Joining method (Saitou and Nei, 1987). The analysis involved 302 nucleotide sequences downloaded from the GenBank database including two representative sequences from each *Mycoplasma* or *Ureaplasma* species and all unclassified species. Our sequences clustered into three significant clusters depicting sequences clustering with *Mycoplasma hominis* strains (Subtree 1), with *Candidatus Mycoplasma girerdii* strains, and strains of novel *Mycoplasma* species (Subtree 2) and with *Ureaplasma* spp. strains (Subtree 3). Accession numbers of the reference sequences used are the following: AB299548.1, AB299549.1, AB558897.1, AB558898.1, AB558899.1, AB576869.1, AB617737.1, AB617738.1, AB680604.1, AB680624.1, AB680625.1, AB680668.1, AB680669.1, AB680678.1, AB680679.1, AB680680.1, AB680681.1, AB680682.1, AB680683.1, AB680684.1, AB680685.1, AB680687.1, AB680688.1, AB680689.1, AB680690.1, AB680691.1, AB680692.1, AB680693.1, AB680694.1, AB725596.1, AB740010.1, AB740012.1, AB758439.1, AB758440.1, AB848713.1, AF001173.1, AF009831.1, AF009832.1, AF042194.1, AF060821.1, AF064062.1, AF125584.1, AF125585.1, AF125587.1, AF125588.1, AF125589.1, AF125592.1, AF125593.1, AF125878.1, AF125879.1, AF132740.1, AF132741.1, AF178676.1, AF212859.1, AF221111.1, AF221112.1, AF221113.1, AF221114.1, AF221115.1, AF221116.1, AF221117.1, AF221118.1, AF221119.1, AF221120.1, AF221121.1, AF261729.1, AF261730.1, AF304323.1, AF304324.1, AF304325.1, AF306346.1, AF538681.1, AF538682.1, AF538683.1, AF538684.1, AF538961.1, AJ002265.1, AJ419900.1, AJ419903.1, AJ419905.1, AM073012.2, AM073013.1, AM073015.1, AM745338.1, AM774638.1, AY050170.1, AY121107.1, AY121108.1, AY150066.1, AY171918.1, AY191226.1, AY366210.1, AY383241.1, AY466443.1, AY529641.1, AY531655.1, AY714305.2, AY756171.1, DQ464424.1, DQ464425.1, DQ641256.1, DQ653410.1, DQ840512.1, DQ840513.1, E02783.1, EF036469.1, EF577506.1, EU646197.1, EU646198.1, EU789558.1, EU789559.1, EU888930.1, FN392885.1, FN392886.1, FN436019.1, FN908083.1, FN908084.1, FN984917.1, GU124613.1, GU124614.1, GU227372.1, GU227388.1, GU227389.1, GU227390.1, GU227391.1, GU227392.1, GU227393.1, GU227394.1, GU227395.1, GU227396.1, GU227397.1, GU227398.1, GU230144.1, GU562823.1, GU569852.1, GU569853.1, GU905011.1, GU905012.1, HM235423.1, HQ634379.1, HQ634380.1, JN214358.1, JN214359.1, JN644767.1, JN644768.1, JN935881.1, JN935892.1, JN935893.1, JQ689949.1, JQ689950.1, JQ897386.1, JQ897387.1, JQ897388.1, KC512403.1, KC512404.1, KF419350.1, L08054.1, L22210.1, L24103.1, L33760.1, L33765.1, M23939.2, M86340.1, NR_024977.1, NR_024978.1, NR_024979.1, NR_024981.1, NR_024982.1, NR_024983.1, NR_024984.1, NR_024985.1, NR_024986.1, NR_024987.1, NR_024988.1, NR_025055.1, NR_025061.1, NR_025062.1, NR_025063.1, NR_025064.1, NR_025065.1, NR_025068.1, NR_025069.1, NR_025070.1, NR_025071.1, NR_025133.1, NR_025134.1, NR_025135.1, NR_025176.1, NR_025177.1, NR_025178.1, NR_025179.1, NR_025180.1, NR_025181.1, NR_025182.1, NR_025183.1, NR_025184.1, NR_025185.1, NR_025186.1, NR_025187.1, NR_025188.1, NR_025896.1, NR_025912.1, NR_025913.1, NR_025914.1, NR_025954.1, NR_025963.1, NR_025964.1, NR_025965.1, NR_025966.1, NR_025968.1, NR_025971.1, NR_025984.1, NR_025985.1, NR_025986.1, NR_025987.1, NR_025988.1, NR_025989.1, NR_026017.1, NR_026034.1, NR_026035.1, NR_026036.1, NR_026037.1, NR_026155.1, NR_029174.1, NR_029175.1, NR_029180.1, NR_029181.1, NR_029183.1, NR_036954.1, NR_037123.1, NR_041844.1, NR_041846.1, NR_042948.1, NR_043138.1, NR_044664.1, NR_044668.1, NR_044673.1, NR_044767.1, NR_044772.1, NR_044811.1, NR_074135.1, NR_074289.1, NR_074301.1, NR_074611.1, NR_074620.1, NR_102477.1, NR_103942.1, NR_104953.1, U02968.1, U04644.1, U04645.1, U04646.1, U04648.1, U04649.1, U04650.1, U04651.1, U04652.1, U04653.1, U04654.1, U04655.1, U04656.1, U09786.1, U09787.1, U09788.1, U15794.1, U15795.1, U16323.1, U16758.1, U16759.1, U16760.1, U19768.1, U22013.1, U22415.1, U26036.1, U26042.1, U26043.1, U26045.1, U26047.1, U26049.1, U26051.1, U26053.1, U26055.1, U29676.1, U44763.1, U44764.1, U44765.1, U44768.1, U44769.1, U44771.1, U56733.1, U58504.1, U58997.1, U67943.1, U67944.1, U67945.1, U67946.1, U83502.1, U83663.1, X00921.1, X62699.1, X76560.1, and Y00149.1.

**Figure 2:**
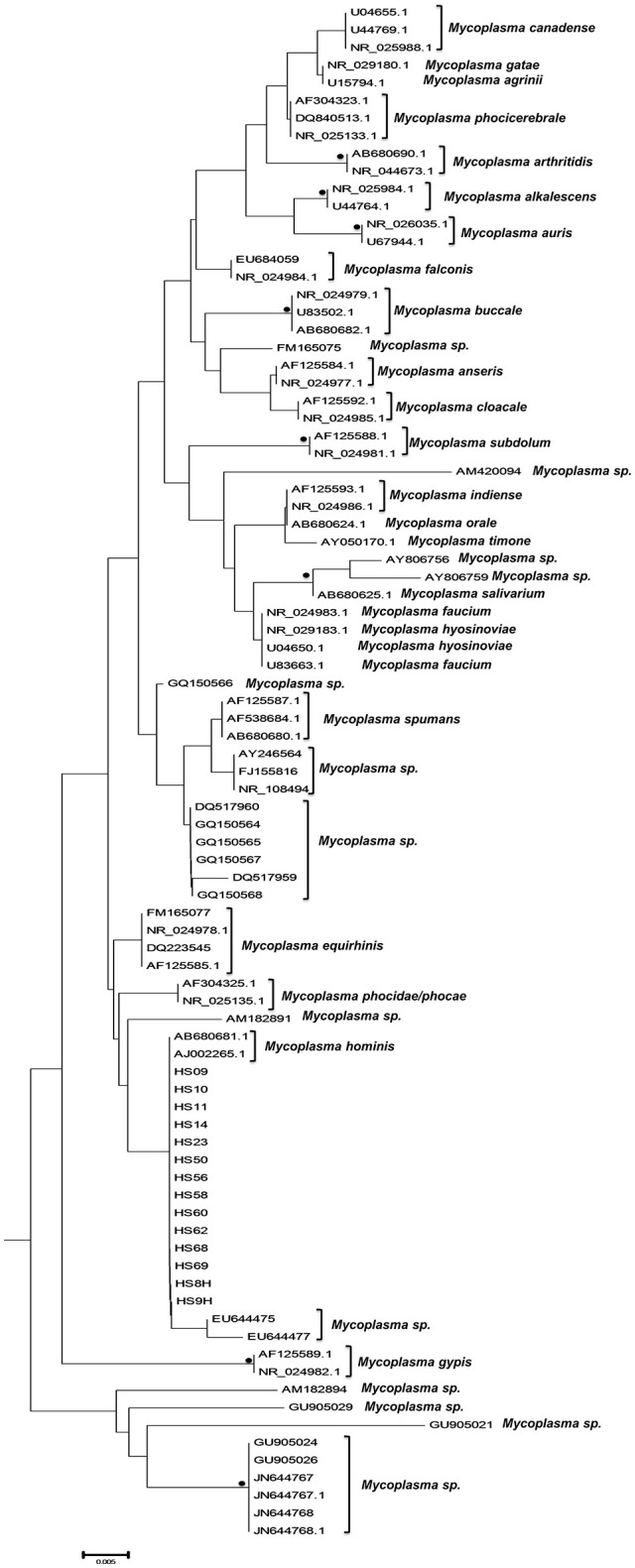
Subtree 1 depicting sequences clustering with *Mycoplasma hominis* strains. Bullets represent significant clustering as indicated by bootstrapping (>75% of permuted trees). The strains of the present study are designated with “HS” and the respective number.

The phylogenetic analysis classified the various *Mycoplasma* species, as well as the correlation between multiple isolates of the same species by constructing phylogenetic trees based on the sequence analysis. 302 *Mycoplasma* and *Ureaplasma* sequences were deployed to sketch the phylogenetic tree (Figure 1). 14 strains (submitted to the GenBank database with the following accession numbers HS09: KY781839, HS10: KY781841, HS11: KY781842, HS14: KY781845, HS23: KY781852, HS50: KY781873, HS56: KY781876, HS58: KY781878, HS60: KY781880, HS62: KY781882, HS68: KY780615, HS69: KY781885, HS8H: KY781838, HS9H: KY781840) were grouped with two *M. hominis* reference strains (accession numbers AB680681.1 and AJ002265.1), whereas, within this cluster, two unclassified mycoplasmal strains were also included (EU644475, EU644477; Figure 2).

**Figure 3:**
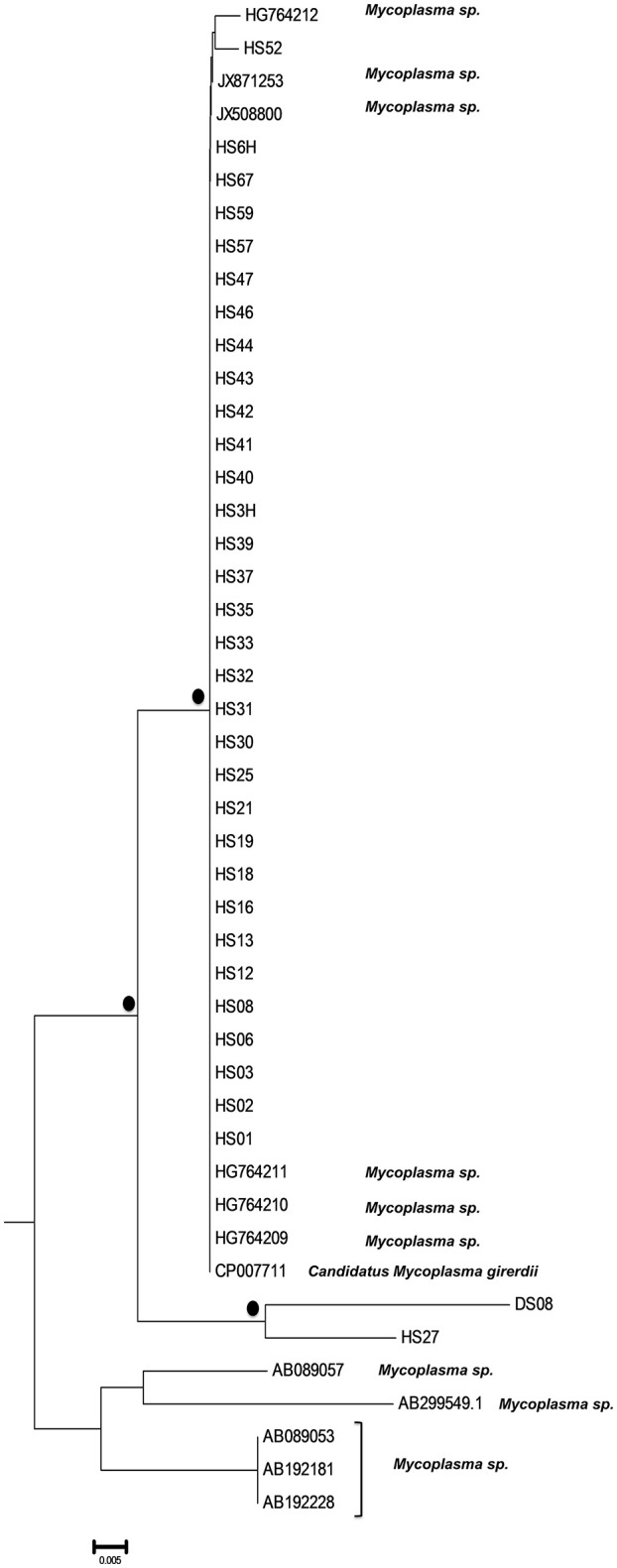
Subtree 2 depicting sequences clustering with *Candidatus Mycoplasma girerdii* strains, and strains of novel *Mycoplasma* species. Bullets represent significant clustering as indicated by bootstrapping (>75% of permuted trees). The strains of the present study are designated with “HS” or “DS” and the respective number.

A bigger group of 34 sequences created a unique cluster with the GenBank accession numbers DS08: KY781830, HS01: KY781831, HS02: KY781832, HS03: KY781833, HS3H: KY781834, HS06: KY781835, HS6H: KY781836, HS08: KY781837, HS12: KY781843, HS13: KY781844, HS16: KY781847, HS18: KY781849, HS19: KY781850, HS21: KY781851, HS25: KY781853, HS27: KY781855, HS30: KY781857, HS31: KY781858, HS32: KY781859, HS33: KY781860, HS35: KY781861, HS37: KY781863, HS39: KY781865, HS40: KY781866, HS41: KY781867, HS42: KY781868, HS43: KY781869, HS44: KY781870, HS46: KY781871, HS47: KY781872, HS52: KY781874, HS57: KY781877, HS59: KY781879, HS67: KY781884 (Figure 3). 32 of them were clustered with six unidentified *Mycoplasma* strains (HG764212, JX871253, JX508800, HG764211, HG746210, HG746209) and with a new species, *Candidatus M. girerdii* (CP007711). The high bootstrap support and the alignment between the sequences of these strains compared to the reference sequence, led to the conclusion that they also belong to the same *Mycoplasm*a species. The remaining strains (DS08, HS27) form a distinct cluster that relates distantly with the *Candidatus M. girerdii* group, suggesting a possible new unidentified *Mycoplasma* species associated with *T. vaginalis* infection.

**Figure 4:**
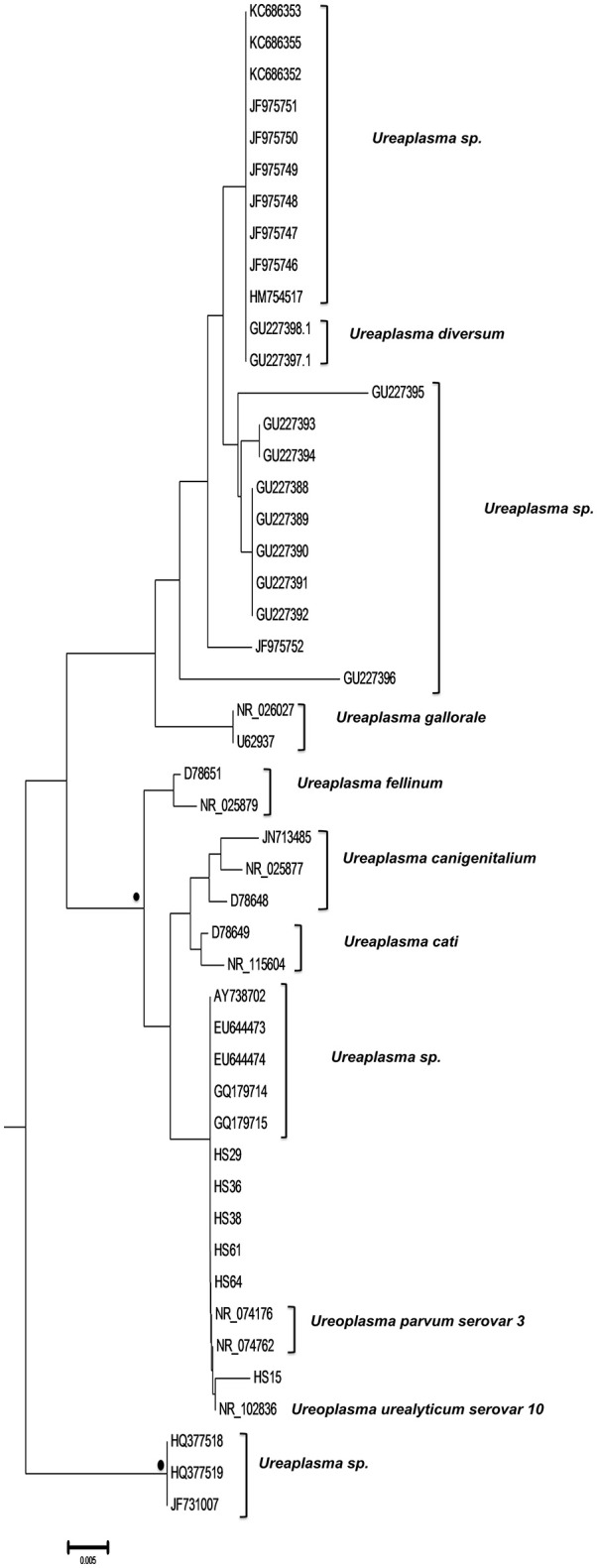
Subtree 3 depicting sequences clustering with *Ureaplasma* spp. strains. Bullets represent significant clustering as indicated by bootstrapping (>75% of permuted trees). The strains of the present study are designated with “HS” and the respective number.

Finally, six strains were clustered with *Ureaplasma* strains (GenBank accession numbers HS15: KY781846, HS29: KY781856, HS36: KY781862, HS38: KY781864, HS61: KY781881, and HS64: KY798518) of which five were associated with 2 *U. parvum* serovar three reference strains (NR074176, NR074762) and one with *U. urealyticum* serovar 10 reference strain (NR102836) (Figure 4).

## Discussion

Aspects regarding the pathogenic bacteria symbiosis with protozoan vectors (entry mechanism, virulence alteration, cell cycle) have paved the way toward deciphering symbiotic relationships though the exact principles remain unknown (Catta-Preta et al., 2015). To this end, the presence of Mollicutes within *T. vaginalis* isolates has been investigated and special interest has been raised regarding the dualism of the beneficial outcome of this co-existence. Are mycoplasmas with the annotated small genome size and the absent cell wall the only ones who benefit from this interplay? To what extent does this symbiosis enhance the existence and favor the virulence of *T. vaginalis*?

Previous studies have shown that the protist tolerates and even favors the growth of the intracellularly located mycoplasmas, which benefit a safe niche protected from the hostile host environment (Dessi et al., 2005). *T. vaginalis* is an exclusively human pathogen, primarily transmitted through sexual contact; thus, all the mycoplasmas and the unidentified species of the present study should be of human origin, most probably acquired by the protozoan through consecutive passages in the various human hosts along the transmission chain of the genital tract infection (Johnston and Mabey, 2008; Harp and Chowdhury, 2011).

According to the *Trichomonadaceae* culture results of our study, only 2% of the *T. vaginalis* strains detected in the vaginal swabs harbor *Mycoplasma/Ureaplasma* spp. implying that the extracellular colonization of the vaginal mucosa might be independent from the intracellular. Since the conventional cultural method is solely suitable for the growth and identification of *M. hominis* and *U. urealyticum*, the extracellular presence of a *Mycoplasma* spp. other than *M. hominis* or *U. urealyticum* in the vaginal epithelium cannot be excluded. In our case, molecular techniques and strain typing have offered a clearer picture on mycoplasmal biology. However, the presence of abundant genes with high diversity pose technical and methodological limitations regarding the PCR and sequencing analysis leading to non-reliable results when it comes for mixed infections (Kanagawa, 2003). Herein, such a limitation would restrict the identification of more than one *Mycoplasma* or *Ureaplasma* species within the same specimen. Next-generation sequencing might contribute in detecting potential *T. vaginalis* co-infections with multiple bacterial species.

The results from the quantitative PCR of the filtrates indicated the absence of *T. vaginalis* DNA contamination by extracellularly located mycoplasmas implying that the *T. vaginalis* rich culture sediments were pure. According to the phylogenetic analysis, the majority of the strains identified belong to species other than *M. hominis*. Both the unclassified *Mycoplasma* strains EU644475 and EU644477 detected in our study, have been previously isolated from amniotic fluid and connected with intra-amniotic infection and inflammation leading to preterm birth (PTB; Han et al., 2009).

The novelty of the current study is the confirmation of the ability of *Candidatus M. girerdii* and other newly reported and previously unknown *Mycoplasma* spp. to survive and multiply within *T. vaginalis*. *Candidatus M. girerdii* has been previously detected in co-infection cases with *T. vaginalis*, as an emerging *Mycoplasma* spp. associated with trichomoniasis. However, there is no evidence to confirm the endosymbiotic relationship of the two pathogens (Martin et al., 2013; Fettweis et al., 2014). *Candidatus M. girerdii* as well as other unknown *Mycoplasma* spp. have been described in association with *T. vaginalis* but only as directly detected in vaginal discharge and not in pure *T. vaginalis* cultures (Martin et al., 2013; Allen-Daniels et al., 2015). Strains HG764209, HG746210, HG746211, HG746212 have been previously isolated as part of the microbiome in low birth-weight infants (Costello et al., 2013), JX871253 has been identified as part of the vaginal microbiome correlating with PTB (Hyman et al., 2014) and JX508800 has been associated with *T. vaginalis* infection as a member of the *Mycoplasma* genus (Martin et al., 2013).

With respect to the *Ureaplasma* spp. most studies have focused on the implication of the bacteria in the antenatal as well as the perinatal period. The absence of reports regarding the symbiosis of *T. vaginalis* with ureaplasmas sets a serious limitation in understanding the possible relationship between the protozoan and the bacteria (van der Schee et al., 2001; Diaz et al., 2010). In this research, we report for the first time the presence of intracellularly hosted ureaplasmas clustered in both known and unknown species. Interestingly, we identified five unclassified *Ureaplasma* spp. strains as intracellularly hosted within *T. vaginalis* isolates of which three have already been reported in the literature and correlated to genital infections as extracellular pathogens. Specifically, AY738702 has been previously isolated from cases of bacterial vaginosis (Oakley et al., 2008), and EU644473 as well as EU644474 have been identified as etiologic agents of intra-amniotic infection and inflammation leading to PTB (Han et al., 2009).

After all, this type of endosymbiosis can trigger challenging-to-handle pathologic phenomena associated with both pathogens involved in the infection. The protozoan-bacterial co-existence with the annotated ability to upregulate the inflammatory process, is reported to be associated with an increased risk for cervical cancer, peripartum complications (chorioamnionitis, PTB, low birth weight), endometritis, pelvic inflammatory disease and complications in women with human immuno-deficiency virus (HIV) infection (Tsai et al., 1995; Huang et al., 2001; Larsen and Hwang, 2010; Djigma et al., 2011; Haggerty and Taylor, 2011; Taylor-Robinson and Lamont, 2011; Thurman and Doncel, 2011; Fraga et al., 2012; Sinkovics, 2012; Fichorova et al., 2013). Another aspect is the clinical importance of the new *Mycoplasma* spp. and the association with specific pathological entities. As supported by the scientific literature, mycoplasma-infected *T. vaginalis* strains present inconsistent results regarding the apparition of metronidazole resistance; in the majority of the studies, no statistically significant association links *T. vaginalis* strains infected by *M. hominis* with metronidazole resistant phenotypes (Xiao et al., 2006; Butler et al., 2010; da Luz Becker et al., 2015). However, drug resistance acquired when unidentified bacteria are hosted in *T. vaginalis* sets an interesting yet important burden in the course of the infection which may complicate the currently applied therapeutic practices and needs further exploration. The role of the different *T. vaginalis* strains in the acquisition or preference of certain bacteria also remains undetermined; however, such a finding would shed light on the clinical impact of this symbiosis within the human host.

As mentioned above, we report for the first time the presence of *T. vaginalis* endosymbionts other than the widely investigated *M. hominis*. Other studies investigating the *T. vaginalis* isolates infection by *M. hominis* usually perform several methodologies to confirm the symbiotic relationship and clarify the clinical implications of this partnership. The most prevailing techniques applied for this reason include (i) immunofluorescence staining assays which differentiate the intracellularly from the extracellularly located mycoplasmas; (ii) microscopy to identify the cellular composition of the internalized mycoplasmas after being endocytosed by *T. vaginalis*; (iii) cytochemistry of acid phosphatase to trace the mycoplasmal degradation products, and (iv) PCR analysis for the mycoplasmal DNA detection within the protozoan cells (Dessi et al., 2005; Vancini and Benchimol, 2008). In our case, the intracellular location of the Mollicutes and the absence of cross-contamination or extracellularly-located species were substantiated by (i) daily passages of the *T. vaginalis* isolates in cultivation media enriched with chloramphenicol, (ii) supernatants filtration and verification of the mycoplasmal DNA absence in the filtered supernatant by quantitative real-time PCR, and (iii) the diversity among the different genera and even within the same species.

One possible limitation of our experimental methodology could be the absence of further evidence supporting the outcomes of this partnership such as the evaluation of the inflammatory process, phagocytosis, and haemolysis following the dual activity of the protozoan and the bacteria (Cirillo et al., 1997; Dessi et al., 2005, 2006; Vancini et al., 2008; Vancini and Benchimol, 2008; Morada et al., 2010; Fiori et al., 2013). Therefore, a future aspect that could follow this study is the confirmation of the endosymbiotic relationship of the protist hosting *M. hominis* as well as the newly identified Mollicutes with the traditional techniques of staining, microscopy, and immunological profiling of the host-pathogen interaction.

## Conclusion

*T. vaginalis* strains isolated from vaginal infection specimens might host intracellular mycoplasmas, which comes in accordance with our findings presenting an equivocal rate of *T. vaginalis* strains negative and positive for hosting Mollicutes. The present study aimed to investigate the existence of intracellularly located bacteria as well as the classification of the *Mycoplasma* and *Ureaplasma* spp. based upon the phylogenetic analysis. The added value of this work is the confirmation of the relatively new species *Candidatus M. girerdii* as a *T. vaginalis* endosymbiont and the first reports on the symbiotic relationship between the protist and the *Ureaplasma* spp. This work lays the foundation for further investigation of the pathobiology of *U. parvum* in trichomoniasis and the role that ureaplasmas play in genital infections and not solely in obstetric infections. Finally, a more sophisticated research strategy is needed to verify whether the novel species identified in this study could play a role as endosymbionts in *T. vaginalis*, act as extracellular commensals or pathogens in human vaginitis. Molecular detection in asymptomatic women and women with non-trichomonas infection could serve as a potent method to answer this question. The symbiotic relationship between *T. vaginalis* and *Mycoplasma/Ureaplasma* spp. discloses novel insights in the “pathogen-to-pathogen” interaction providing a paradigm in “microbial socialization” where “unity is strength.”

## Author contributions

AI, MT, and SC contributed to the conception and design of the study, data analysis and interpretation and drafted the manuscript. EM was in charge of the sequence and phylogenetic analyses. KT included patients, and critically reviewed the manuscript. AB drafted and critically reviewed the manuscript. PP, VI, and MM carried out the laboratory work of the study, contributed to the data collection and analysis, and critically reviewed the manuscript. All authors have contributed intellectually during the writing process, have read the final manuscript and approved the contents.

### Conflict of interest statement

The authors declare that the research was conducted in the absence of any commercial or financial relationships that could be construed as a potential conflict of interest.
